# Preserving the human character in an inhuman situation: the contributions and meanings of military soul care in the Swedish armed forces

**DOI:** 10.3389/fpsyt.2025.1693657

**Published:** 2025-11-18

**Authors:** Jan Grimell

**Affiliations:** Department of Sociology, Faculty of Social Sciences, Umeå University, Umeå, Sweden

**Keywords:** military chaplain, military soul care, spiritual care, existential, mental health, human character, moral injury

## Abstract

Military chaplains (MCs) are trained to address aspects of health beyond the remit of mental health professionals, namely the spiritual/religious and existential dimensions of service members’ mental health, within the biopsychosocial-spiritual (BPSS) model. Models of military spiritual care differ, and in Sweden this is termed Military Soul Care (militär själavård), regulated through the ACCES framework (Advisory role; Commander and Crisis support; Ceremonies; Education; Soul care conversations in absolute secrecy). The study aimed to explore the contribution and meaning that MCs attribute to Military Soul Care in the Armed Forces. A qualitative questionnaire was employed, extending earlier Swedish research while reaching broader scope and depth. Fifty MCs participated. Using an inductive approach, three overarching themes emerged: Preserving the human character in an inhuman situation, Sustaining ethical and moral dimensions and reflections in a military context and, Framing cultural coherence to assist meaning-making in a military context. While these findings resonate with the Swedish Armed Forces’ understanding and purpose of Military Soul Care, they highlight additional emphases that deepen the comprehension of its role. Recent research from the war in Ukraine underscores how sustaining the moral character of military personnel is vital, as the relentless brutality of war erodes the moral frameworks underpinning civilian identities and contexts of peace. This aligns closely with the concept of moral injury. Accordingly, the contribution and meaning of Military Soul Care should be recognized as extending far beyond a spiritual/religious function, constituting a holistic approach to mental health within the BPSS model. The Swedish model is existential rather than spiritual or religious, and inclusive rather than exclusive. As it is culturally embedded within specific Swedish traditions, frameworks, and understandings, it does not satisfactorily align with concepts such as ‘spiritual’ and ‘religious’. Future research is especially encouraged on sustaining the human character and advancing a culturally sensitive approach to military chaplaincy.

## Introduction

Military chaplains (from hereon MCs), whether ordained or not, are present in an organized capacity within most Western armed forces (1). Their specific tasks, roles, and modes of conduct vary both across and within different military organizations (2). Nevertheless, their professional function can generally be characterized as providing forms of expertise, competence, and experience that the military—including the fields of military psychiatry and psychology—does not itself possess (3–5). At the same time, the military regards these contributions as important, or even crucial, for supporting the mental health of personnel before, during, and after deployment (6). Such contributions encompass a range of domains. These include, but are not limited to, managing and addressing death in military contexts in a culturally dignified and appropriate manner; facilitating and supporting processes of grief; delivering education in ethics and moral reasoning; serving in advisory roles to commanders and decision-makers; and offering spiritual and pastoral care in relation to suffering, moral conflict, and questions of existential meaning (2, 5, 7, 8).

While military medical personnel have a distinct role in assessing and evaluating the mental health, fitness, and capacity of service members to carry out their missions and duties—and are therefore subject to mandatory reporting requirements—MCs fulfil an entirely different function within the organization (4, 9, 10). Among other things, they can serve as a *safety release valve* for military personnel, providing a trusted space in which individuals can share their innermost thoughts and feelings, protected by confidentiality—regarded by some MCs as a matter of absolute secrecy—without risk of consequences for their ability to serve. Both the principle of secrecy and the practice of confession illustrate examples of a *safe* sp*ace* for disclosures of all kinds, offered by someone who possesses military cultural competence, shares the lived experience of wearing the uniform, and holds expertise in engaging with issues of moral suffering, moral transgressions, guilt, shame, reconciliation, forgiveness, hope, and meaning (2, 11–14).

### Moral injury and MCs

Closely related, and of particular importance, is the emerging role of chaplains in connection with the concept of *moral injury* (3, 7, 12, 15, 16). Their involvement has opened new avenues for understanding and addressing complex moral and existential challenges that may or may not co-occur with clinical conditions such as post-traumatic stress disorder (PTSD), within the framework of medical and psychological care for service members and veterans (17, 18). This convergence of spiritual and pastoral care with clinical treatment constitutes a novel and increasingly recognized dimension of comprehensive care for military personnel and veterans (12, 15, 16, 19, 20).

### Aim

The aim of this article is to examine the contributions and meanings of the Swedish model of military chaplaincy support—known as Military Soul Care—beyond the official statements of the Swedish Armed Forces. The purpose is to gain a deeper understanding of Military Soul Care from the perspective of the practitioners themselves, exploring how they perceive, interpret, and prefer to describe their practices at the operational, *boots-on-the-ground* level. The empirical material derives from a qualitative survey conducted in March 2025 among 50 Swedish MCs, focusing on their experiences of Military Soul Care.

The article proceeds by outlining the roles of MCs from a global perspective before turning to a more in-depth examination within a Swedish context.

## A global perspective on the roles and theoretical frameworks of MCs in active conflict

The missions and functions of military MCs differ significantly across national armed forces. Recent scholarship underscores that societal and cultural norms, legal frameworks, canon law, and military regulatory documents all influence how their roles are shaped and operationalized (2). As a result, it is both challenging and methodologically problematic to discuss MCs’ roles and responsibilities in too sweeping or generalized terms.

### Ministry through presence

Generally speaking, MCs engage with the spiritual or religious dimensions of military personnel, as well as with their secular or humanistic needs. They typically remain embedded with the troops and practice what has been described as a *ministry through presence*, meaning they maintain a consistent, relational proximity to service members (21–25). This involves being available in the everyday realities of military life—listening, offering guidance, and supporting individuals in times of questioning, struggle, and suffering. MCs are often trained to provide attentive, empathic support, offering interpretation and moral encouragement while safeguarding service members’ stories, emotions, and challenges under strict confidentiality. This confidentiality, as already noted, is foundational to their function, ensuring absolute privacy in pastoral encounters.

### Military cultural understanding

Military chaplains usually possess a high degree of cultural literacy within the military environment (4, 10), and some even hold military rank. This cultural understanding enables them to serve alongside service members, sharing their environment, adopting military customs, and embodying the values and ethos of the armed forces, often symbolized by the wearing of the uniform. Their embedded presence within the military organization allows them to develop deep contextual awareness and relational trust.

### Practical and formal duties

MCs perform a range of duties that may include pastoral and spiritual care, educational roles, ceremonial responsibilities, advisory functions to commanders, and crisis or trauma support. These responsibilities vary internationally, reflecting how chaplaincy services are structured in response to each nation’s historical trajectory, religious landscape, cultural context, and military and civil legal systems. Consequently, MCs in one country may undertake responsibilities that do not exist in another. Many chaplains also facilitate rituals, commemorations, reflective practices, and activities intended to support moral and spiritual formation within the armed forces. Rituals—whether religious, cultural, or symbolic—can cultivate resilience, restore meaning, and offer hope in moments of anxiety, loss, or uncertainty (2).

### War and MCs

Recent research from the war in Ukraine has demonstrated that a substantial component of wartime military chaplaincy involves sustaining the moral character and compass of military personnel (26, 27). This is necessitated by the fact that the relentless and unremitting brutality of war steadily erodes and dismantles the moral framework that had previously shaped civilian identities and the everyday contexts of peace—frameworks that no longer exist within the realities of armed conflict. Ukrainian MCs—arguably the most experienced in the world today in caring for and supporting frontline personnel in a full-scale war—have underscored the critical importance of persistently engaging with moral and ethical issues. Their aim is to counteract war’s corrosive effect on soldiers’ character, an erosion that, if left unchecked, can impair operational effectiveness, diminish judgment, foster recklessness, and contribute to wartime transgressions that are profoundly difficult to live with (26, 27).

### Varieties of theoretical frameworks

A number of established conceptual frameworks exist for articulating the forms of care provided by MCs across different contexts. These frameworks may be adapted to the military environment by applying the prefix *military*. However, such concepts are not universally applicable; they are shaped by distinct religious traditions, institutional histories, and national defence structures, each of which may employ its own terms and interpretive paradigms. The purpose of the following overview is not to offer an exhaustive taxonomy, but rather to introduce several key frameworks and to illustrate how they correspond—or fail to correspond—to the Swedish model.

### Military pastoral care

The first framework is *pastoral care*, a more narrowly defined term traditionally employed within religious communities that are organized around ordained or authorised clergy such as priests and pastors (28–30). In these traditions, military pastoral care is rooted in particular ecclesiastical heritages and shaped by their theologies, liturgical practices, and canonical structures (e.g., Protestant, Catholic, Orthodox).

### Military spiritual care

The second framework, *spiritual care*, is broader and more inclusive in scope. It can be applied across diverse religious and spiritual traditions and may include both ordained leaders and recognised lay representatives (15, 31, 32). In the military environment, *military* sp*iritual care* refers to the support, guidance, and counselling of service members, veterans, and units by chaplains who may be religious ministers or authorised representatives, regardless of ordination status. This framework is internationally acknowledged and widely employed (3, 5, 7, 15, 32).

It is also important to note that the concept of *spirituality* itself is contested and subject to ongoing debate. The term is utilised by theological disciplines, religious traditions grounded in God, the Trinity, the Spirit, or Allah, by faiths with multiple deities or transcendent powers, and by secular and humanist contexts that likewise seek to define spirituality within their practices.

### Military spiritual fitness

Military spiritual care can readily be aligned with open and inclusive definitions of spirituality, such as the pursuit of meaning and purpose in life (33, 34). This aligns with the concept of *military* sp*iritual fitness* (35), which has gained prominence in the United States.

### Military existential care

Depending on how spirituality is defined—and recognising the challenges of translating such definitions across cultural contexts—there may be a need to conceptualise care from an existential perspective. This approach, referred to as *existential care*, becomes relevant in settings where religion and spirituality do not align neatly with conventional understandings of pastoral or spiritual care (36). Alternatively, a hybrid approach combining existential and spiritual care elements may be considered (37). While the term *existential care* has not yet been fully established conceptually, it offers a promising framework for exploring alternative models of chaplaincy in contexts where traditional categories do not accurately reflect cultural realities or chaplaincy practice (2).

### Military Soul Care

The final framework originates entirely from the Swedish context and does not readily correspond with either pastoral care or spiritual care. It resonates most closely with an existential approach. This brings the discussion to the Swedish model by first situating it within its cultural and contextual roots.

## The swedish context

Sweden is widely recognized as a highly secularized and pluralistic (38). However, when moving beyond the metrics typically employed in surveys such as the *World Values Survey* (39), one finds a complex cultural and historical tradition that resists such simplified categorizations (11, 40, 41). Until the year 2000, Sweden maintained one of the most extensive state church systems in the world (42). Although the Church of Sweden was formally separated from the state at that time, it continues to be governed through traditional political representation, with national, regional, and local elections (11). Despite this so-called *divorce*, long-standing institutional, cultural, and ritual structures remain deeply embedded in Swedish society (43). For example, clergy of the Church of Sweden have served continuously in the Swedish Armed Forces since the 16th century (44).

Today, more than half of the population—approximately 5.4 million individuals—remain members of the Church of Sweden (45), with membership numbers even increasing. In addition to the Church of Sweden, a diverse range of other religious communities are active in Sweden, including Free Churches, and Catholic, Orthodox, Muslim, Buddhist, Hindu, and Jewish congregations. These contribute to a more nuanced picture of Swedish religiosity than the one suggested by broad claims of secularization. Nevertheless, the Church of Sweden holds a unique position among faith communities, having shaped the cultural, social, and political development of the nation since the Reformation. It continues to maintain and transmit traditions, rituals, and cultural practices that can be seen as constitutive elements of Swedish culture and identity (11, 40, 43).

### Soul care as a church-specific practice that has resisted psychologization

In the Swedish context, it is noteworthy that the psychologization of pastoral practice, which took place in North America during the mid-20th century, did not take root. In the United States, this process exerted a strong influence on conceptual developments within pastoral psychology, clinical pastoral education (CPE), and related professional requirements. When pastors were integrated into the healthcare sector during its modernization beginning around 1940, concepts and terminology were adapted to the prevailing secular context. Classical expressions such as *the cure of souls* (46) were neutralized and increasingly replaced by the language of secular psychology. Instead of *the cure of souls*, the pioneering figures in pastoral psychology, Cabot and Dicks (47) and later Dicks (48), introduced the concepts *pastoral work* and *pastoral counseling*, which remain dominant today (49). The CPE movement subsequently spread to Europe through the Netherlands and Germany, and it gained traction in neighboring countries such as Norway and Finland, where it became a formalized and recognized part of pastoral practice (50, 51).

Sweden (and Denmark), however, represents an exception in this respect. The influence of CPE did not take hold, in part because it was perceived as incongruent with the theological orientation and education, and intellectual currents of Swedish practical theology in the postwar period (51; see also 52, Special Issue on Chaplaincy in Northern Europe). As a result, clergy (and deacons) and chaplains in the Swedish church tradition continue to practice *soul care* under the premise that all human beings possess a soul that faces existential challenges and therefore requires care. This approach can be described as more existential and generic, rather than confessional or narrowly ecclesial.

While the Church of Sweden’s practice of *soul care* is closely related, it cannot be directly equated with Military Soul Care. The latter represents a distinct, military-specific framework in which *soul care* constitutes only a minor component within a broader range of responsibilities, as delineated in the Military Soul Care framework (11) according to the ACCES acronym presented below.

### The Swedish Armed Forces’ understanding and purpose of Military Soul Care

Within the Swedish Armed Forces, there is a profound awareness that military personnel require more than physical training alone in order to defend the Swedish nation and uphold its values. Military Soul Care is therefore tasked with strengthening a sense of cultural coherence, thereby enhancing individual performance, mental resilience, and moral fortitude. A strong moral and ethical foundation is regarded as indispensable for the ability to act and make decisions under pressure and in critical situations (53).

The purpose of Military Soul Care is to foster spiritual balance and existential health, while also increasing individual resilience in crises and war. This is considered an important dimension of Sweden’s psychological defense and national will to resist. Military Soul Care is inclusive and offered unconditionally, regardless of religious affiliation or personal belief. It promotes human dignity, equality, and the value foundation of the Swedish Armed Forces (53).

Military Soul Care is organized around five overarching tasks, summarized in the acronym ACCES:

- Advisory role (to commanders in military contexts as well as to the Church of Sweden regarding issues of war preparedness, etc.)
- Commander and Crisis support
- Ceremonies (including field sermons, prayers, memorials, and related rituals)
- Education (ethics and moral training, instruction on war graves, etc.)
- Soul care conversations (offered in absolute confidentiality)

Military Soul Care finds its locus within the Armed Forces’ doctrinal pillar of *moral factors*, thereby contributing to the overall capacity for warfare. Moral factors encompass human qualities such as willpower, courage, resilience, esprit, leadership, education, exercise, training, and ethics (54).

In summary, the Swedish framework of Military Soul Care is structured through ACCES, situated within a distinctly Swedish cultural sense of coherence. It is rooted in the doctrinal pillar of *moral factors*, which serve to enhance the overall capacity for warfare. Its aim is to strengthen moral character and compass, foster spiritual balance and existential health, and thereby increase the inner resilience and capacity of military personnel to carry out their duties in times of war. MCs draw upon their comprehensive theological and ecclesial resources, their pastoral experience, and their nuanced understanding of military culture, training, and operational realities in order to implement an existentially sensitive Military Soul Care approach in their work with ACCES.

### Priests serving as MCs

Within the Swedish Armed Forces, MCs are, in principle, ordained priests of the Church of Sweden. This reflects a range of factors, including the high academic theological requirements for appointment as a MC, the need for alignment with the Armed Forces’ value foundation ​​(e.g., LTGB rights), the expectation of substantial pastoral experience and parish employment, as well as stringent security clearance procedures, regulations, cultural norms, and traditions (11). In practice, these conditions mean that, in principle, only priests from the Church of Sweden are appointed to serve in this capacity.

## Method

The material on which this article builds derives from a qualitative study of Swedish MCs, carried out in March 2025 as part of a project commissioned by the Chief of Chaplains of the Swedish Armed Forces. The purpose of the study was to broaden and deepen knowledge about Swedish MCs in a contemporary setting. As little qualitative research has been conducted on this professional group in today’s context, a qualitative design was considered particularly appropriate.

The study was reviewed and approved by the Swedish Ethical Review Authority (Reference number: 2024-08417-01).

### Data collection

To extend earlier Swedish research and simultaneously obtain greater scope and depth, a qualitative questionnaire was chosen as the method of data collection (55, 56). This design allowed the inclusion of a comparatively large number of participants while still providing the kind of nuanced and open responses normally associated with qualitative interviews (55, 56). The approach offered both a broad overview of MCs’ experiences and an opportunity for participants to articulate their perspectives freely and in their own words. Importantly, the questionnaire was entirely anonymous, thereby lowering potential barriers to sharing personal reflections and experiences.

### Questionnaire focus

The full questionnaire comprised 31 questions distributed across nine pages and included open-ended items. A thematic section titled *Military Soul Care* contained questions specifically designed to explore participants’ perspectives on its contributions and meanings. The analysis presented in this article is based on the following five questions:

- In your view, what does Military Soul Care contribute?
- Have you, in your role as MC, worked with value based exercises and or education in ethics and morality (please describe)?
- How familiar are you with the concept of *moral injury*?
- Is it a concept you have used in Military Soul Care (if yes, please describe as far as possible)?
- What significance do you ascribe to the MC’s duty of absolute secrecy?

The first question has formed the principal focus of analysis in this article, while the subsequent four have assumed a secondary yet complementary function, contributing to a more nuanced understanding of Military Soul Care in dimensions of particular significance to the framework.

### Implementation of the study

The questionnaire was distributed during the training days for Swedish MCs, held on March 12 to 13, 2025, at the Armed Forces Headquarters in Stockholm. Because the use of electronic devices such as computers and mobile phones was not permitted in the premises, the questionnaire was administered in paper format. The study was introduced orally, after which participants received written information and a consent form. Participation was entirely voluntary, and those who did not wish to take part were free to decline. Participants were given the option to leave the room and complete the questionnaire in several areas outside the lecture hall. The vast majority chose to leave the room, which ensured ample opportunity to abstain from participation and return once the allotted time had passed.

Respondents were given 1 hour and 40 minutes to complete the questionnaire, corresponding to the length of a relatively long research interview. To ensure anonymity, consent forms were collected separately from the completed questionnaires, making it impossible to trace individual responses back to specific participants.

### Swedish military chaplains

In Sweden, MCs serve in several distinct capacities. A common feature is that, with only a few exceptions, they are ordained Lutheran priests within the Church of Sweden.

One group consists of priests formally employed by the Church of Sweden who, alongside their regular parish responsibilities, also serve as MCs at regiments, air bases, naval stations, and garrisons within their pastoral districts. Typically, between 20 to 75 percent of their time is devoted to military duties, with the remainder spent in parish work. For simplicity, these respondents are hereafter referred to as *Regiments, Air Bases, Naval Stations, and Garrisons MCs*.

Another group comprises chaplains attached to Home Guard battalions. These battalions consist of part time military personnel with civilian professions who can be rapidly mobilized. MCs in this category work full time as parish priests and in addition serve approximately 8 to 10 compulsory days per year in their assigned battalions. These days are not normally included in their parish employment, but rather fall under the Home Guard, that is, the Swedish Armed Forces. These respondents are hereafter referred to as *Home Guard Battalion MCs*.

A third category consists of chaplains serving at the regional command level. They are attached to one of Sweden’s five military regions, North, Central, West, South, and Gotland. Regional staffs lead operations during national crises and provide civil support, and in this case the employer is the Armed Forces.

Beyond these three main categories, there are exceptions and extended roles (see 57). Furthermore, the Chief of Chaplains commissions regular continuing education days for MCs across categories.

Typically, a Swedish MC has served several years as a parish priest before becoming eligible for military chaplaincy. They often have substantial experience in soul care and counseling. Some also have military backgrounds through conscription or officer training. Certain military courses can be provided as part of the chaplaincy role (see 11, 57).

### Participants

In total, 50 individuals took part in the study, representing somewhat less than half of the steadily growing body of Swedish MCs.

The group comprised 32 men and 18 women. Most were between the ages of 35 and 55, with a few younger than 35 and a few older than 55. Of these participants, 17 served as *Regiments, Air Bases, Naval Stations, and Garrisons MCs*, 24 as *Home Guard Battalion MCs*, 3 held sensitive positions not specified for ethical reasons, and 6 chose not to disclose their current positions due to anonymity concerns.

Career trajectories varied considerably. Many had served in multiple chaplaincy roles, with 21 reporting experience from several different positions. Eleven participants had completed one or more international deployments as MCs. Length of service ranged from newly appointed chaplains to those with 30 years of experience, with an average of 8.75 years, suggesting a highly experienced cohort overall.

To safeguard anonymity, neither gender nor years of service are reported alongside excerpts, as combining such details might risk revealing individual identities. The same principle applies to other potentially identifying information, such as particular deployments, ranks, duty stations, or events, which has been omitted or anonymized when necessary. This was especially important given that participants completed the questionnaire during the same gathering, although they had the option of filling it out in another location.

### Analysis of qualitative data

The completed questionnaires were transcribed into Word documents, with each file corresponding to one of the open ended questions. These documents were then imported into Atlas.ti for coding and analysis. A thematic analysis with an inductive approach (58, 59) was conducted in two main steps, first initial coding, and second the organization of codes into broader thematic structures.

In the initial stage, specific observations were identified and labeled, always in relation to the three guiding questions, namely what Military Soul Care contributes according to the MCs, whether they had worked with value based exercises and or education in ethics and morality, and the significance of absolute secrecy. Coding was thus carried out in close connection with the empirical material. For instance, a response such as “To preserve and strengthen one’s humanity” was coded as *preserving or strengthening humanity*, while “It contributes to the creation of meaning and context” was coded as *meaning and coherence*. In this way, the first analytic step remained closely tied to the original content.

This resulted in a large number of individual codes which, in order to be presented with meaning and clarity, needed to be organized into broader groups. This exemplifies the inductive process, in which a substantial body of material is coded openly and freely while remaining closely anchored to the empirical data, and is subsequently structured into larger groups to enhance both meaning and clarity (58, 59).

The individual codes were then clustered into broader and more abstract code families, the term used in Atlas.ti, in order to support a meaningful thematic analysis of the contributions and meanings of Military Soul Care in the military setting. The logic underlying the creation and naming of the families was based on ensuring a clear correspondence between the individual codes and the broader code families; likewise, the code families were expected to be reflected in the individual codes. There was, therefore, a clear interplay and reciprocal relationship between these two levels. Moving from this micro level to higher order thematic categories inevitably meant a certain loss of nuance, but it was a necessary step in order to present a coherent and analytically valuable interpretation of the data.

The following three code families, which also serve as categories, structured the subcodes:

- Preserving the human character in an inhuman situation
- Sustaining ethical and moral dimensions and reflections in a military context
- Framing cultural coherence to assist meaning-making

The analysis that follows employs these categories as headings, weaving the empirical material together and linking back to it through excerpts from the coding.

## Analysis

The findings clearly show that MCs’ own description of Military Soul Care broadly resonates with the Armed Forces’ understanding and purpose of the MCs’ presence and function within the military organization. Namely, to contribute—through their particular expertise, experience, and capacity—in strengthening the warfighting ability of military personnel by supporting and assisting the spiritual balance, existential health, mental resilience, moral fortitude, and the ethical and value foundation of the Armed Forces through an all-encompassing approach. At the same time, most participants expressed this contribution in their own individual and unique ways, marked by qualitative nuances and variations.

### Preserving the human character in an inhuman situation

Roughly half of the participants explicitly emphasized the safeguarding of the human dimension in an inhuman situation as the principal contribution of Military Soul Care. This was the theme most frequently highlighted, voiced in a near-unanimous chorus. In essence, it reflected an awareness that the chaos of war threatens to dissolve what may be understood as ‘the human’—namely, moral character, ethical integrity, and the capacity to distinguish right from wrong while sustaining meaningful civilian identities within a military context. These qualities, normally upheld in ordinary circumstances, risk erosion and dissolution in prolonged conflict, combat, and war.

Such erosion of what constitutes the human can lead military personnel—or commanders—to lose their moral compass, become reckless, and pose a danger both to themselves and to their units. It can also result in decisions or actions with harmful consequences at multiple levels. At the individual level, this may mean struggling to live with one’s actions afterwards, or even facing court-martial. At the tactical level, it may involve, for instance, the killing of prisoners of war, which could deprive the force of intelligence crucial for understanding enemy maneuvers, tactics, positions, or technology. At the operational level, a decline in the moral character of personnel or units can diminish operational resources and effectiveness. At the level of the armed forces or the nation as a whole, if a military force is perceived as violating international law and conventions, the consequences may include sanctions and external pressure, difficulties in securing support from friendly nations, and the risk of legal repercussions for political leaders and senior commanders.

Thus, the preservation of the human was described by MCs in various ways as a significant contribution of their work. The following examples illustrate how several MCs articulated this contribution, based on their own understanding of what Military Soul Care provides:

Participant 9 (Home Guard Battalion MC) described the contribution and deeper meaning in terms of humanity as:

“To preserve and strengthen one’s humanity. To reflect on life. Preserving the human, even in the enemy. Strengthening the individual.”

Participant 10 (Home Guard Battalion MC) emphasized the safeguarding of humanity as:

“To safeguard the individual’s humanity, and to contribute to the journey from the inhuman back to the human.”

Participant 15 (Home Guard Battalion MC) highlighted the preservation of the human within the collective dimension of military life, explaining it as:

“To strengthen the unit’s collective capacity by equipping individual soldiers in such a way that they remain whole as human beings, even when the demands and chaos of war subject them to severe trials.”

Participant 16 (Home Guard Battalion MC) conveyed the human significance in simple but powerful terms as:

“That the person under pressure remains human, even when the strain intensifies.”

Participant 20 (Home Guard Battalion MC) pointed to the importance of humanity in reintegration after service, stating:

“To ensure that people remain human even after serving as soldiers, and to help individuals recognize themselves again after a mission.”

Participant 31 (Regiments, Air Bases, Naval Stations, and Garrisons MC) stressed the preservation of human dignity in war, noting:

“To safeguard human dignity in war—including that of the enemy—as a counterweight to a marginal and materialistic view of the world/war. To ensure that, amidst the horrors of war, the military continues to embody the society it is meant to defend.”

Participant 33 (Regiments, Air Bases, Naval Stations, and Garrisons MC) underscored the risk of losing humanity within the uniform, observing:

“To safeguard the humanity within the uniform. To ensure that the soldier’s humanity is not lost.”

Participant 45 (preferred not to state their current position) focused on the fundamental human need to protect dignity, phrasing it as:

“To uphold and preserve human dignity in a difficult situation.”

Participant 46 (preferred not to state their current position) emphasized the rediscovery of the human self, explaining it as:

“To help/support people within the Armed Forces so that they do not lose sight of, and can rediscover, the human being behind the uniform.”

Participant 48 (preferred not to state their current position) situated the safeguarding of humanity within doctrine, describing it as:

“Military Soul Care is grounded in the moral pillar of the Armed Forces’ doctrine. Its purpose is, through conversation, support, and guidance, to be present for the individual as well as the group, in order to sustain human dignity, existential health, and identity in both peace and wartime.”

As reflected in the accounts of the MCs, what constitutes a human being—and what it truly means to be human—emanates from what we have learned within the society in which we live: a culture that shapes meaningful identities grounded in morals and norms, enabling the individual to organize, navigate, and make sense of life. In a parallel way, the military community provides its service members with corresponding inner structures—identity, meaning, morals, and norms—that likewise support them in organizing, navigating, and making sense of their military existence.

Thus, the preservation and safeguarding of what was understood to constitute the human and human dignity, before, during, and after deployment, emerged in the study as the most frequently emphasized contribution according to the accounts of the MCs. The opposite of this, which the MCs sought at all costs to prevent, was the dangerous process of dehumanization, directed both toward others and toward oneself. When the human dimension is lost in wartime, research has demonstrated that the consequences are deeply damaging, undermining decision making, resilience, endurance, and motivation (60, 61).

To safeguard the human in military personnel, MCs drew upon extensive expertise, experience, and capacity. This emanated from:

- a centuries-old church tradition with its profound understanding of the caring of human souls—and in particular military souls,
- long experience as parish priests working with people in crisis and grief,
- absolute secrecy, unanimously regarded as fundamental to their mission, providing a vital release valve that allows military personnel to express their innermost thoughts and feelings without fear of disclosure,
- their role as dialogue partners with deep existential expertise,
- theology, tradition, ritual, ceremony, and narrative, offering meaning and interpretive frameworks from existential, spiritual, and cultural perspectives,
- and a specific understanding of military culture.

Together, these resources enabled MCs to support military personnel and to provide interpretive keys for understanding what it means to be human in a military environment, as well as what is at stake when the human dimension is compromised. Ultimately, this relates directly to service members’ decision-making ability, resilience, motivation, and warfighting capacity.

What it means to be human, according to the participants, is an empirical question that exceeds the methodological limitations of the present study. Addressing such a question would require an interview-based research design that enables a profound exploratory inquiry. However, drawing on research concerning MCs in Ukraine, the human condition may be understood as constituted through ethical, moral, and identity-forming processes cultivated within a functioning society characterized by peace, mutual recognition, and dialogical engagement (26, 27). Armed conflict erodes these foundations and risks precipitating an ontological disintegration of the human person, reducing the individual to what has been termed a “war animal” (26, p. 8) In this view, it is the inhumane condition—that is, the dissolution of socially embedded ethical and moral structures, and thereby of stable identity—that serves as the enabling ground for such a transformation (27).

A working definition of an inhumane situation in the context of armed conflict may therefore be understood as a condition that destabilizes or dissolves the ethics, morality, and identities that societies in peacetime consider meaningful, virtuous, and conducive to human flourishing. This suggests that armed conflicts generally engender inhumane conditions. Yet such conditions are not absolute; they are dimensional, contingent upon the character, intensity, and duration of the conflict, as well as the extent to which the fundamental laws and norms of armed engagement are upheld or violated.

### Sustaining ethical and moral dimensions and reflections in a military context

The overarching, self-described theme of preserving the human may at times appear somewhat abstract in the accounts of Military Soul Care. Moreover, the expression *human*, both linguistically and conceptually, reflected the theological and existential mindset of the MCs. By contrast, the second major contribution was more concrete and concerned, in explicit terms, ethics and morality.

It should also be emphasized that a majority of the MCs had worked with value-based exercises, foundational values training, and various forms of education in ethics, morality, and ethical dilemmas within a military context, which resonates with education in ACCES. In many ways, this is closely connected to the preservation of the human in an inhuman situation, that is, the rules, norms, beliefs, and perceptions of right and wrong that constitute what it means to be human in functioning social relations and societies. At the same time, this represented, both analytically and thematically, a much more specific and distinct contribution.

MCs highlighted that Military Soul Care fostered a deeper understanding, insight, and capacity to morally, ethically, and reflectively engage with—and manage—oneself in the corrosive process that deployment and armed conflict can entail for military personnel. This was illustrated by participants in the following ways regarding what they perceived Military Soul Care contributed to:

Participant 9 (Home Guard Battalion MC) described it simply:

“A cultivating sound moral and ethical habits.”

Participant 17 (Home Guard Battalion MC) emphasized:

“A possibility to recalibrate one’s moral compass, again and again.”

Participant 22 (Home Guard Battalion MC) observed that Military Soul Care contributes:

“To our ability as human beings in a war situation to remain ethical, moral, and to follow the UN’s human rights in war.”

Participant 29 (Regiments, Air Bases, Naval Stations, and Garrisons MC) spoke of it in terms of:

“Moral, spiritual, and religious integrity and identity; not only preserving but also deepening the inner compass in a challenging and chaotic environment.”

Participant 34 (Regiments, Air Bases, Naval Stations, and Garrisons MC) highlighted:

“The raising of ethics and morality, as well as value foundations.”

Participant 35 (Regiments, Air Bases, Naval Stations, and Garrisons MC) emphasized the importance of:

“Working with ethical issues in educational settings.”

Participant 37 (Regiments, Air Bases, Naval Stations, and Garrisons MC) articulated it in operational terms:

“Strengthening moral capacity in the context of conflict. Enabling people to perform their tasks more effectively, especially in armed attack, but also during live operations, exercises, and similar contexts. Helping soldiers and sailors to feel better spiritually and mentally, fostering moral and ethical awareness, and sustaining human compassion despite war, exercises, or real missions.”

Participant 40 (Regiments, Air Bases, Naval Stations, and Garrisons MC) described it as:

“A reminder of what is right and wrong, of good and evil—that is, morality.”

Participant 45 (preferred not to state their current position) concluded:

“Creating the conditions for sound ethics and morality.”

The sustaining of morality and ethics could be both planned and formal—through education, rituals, ceremonies, commander support, or the advisory role of the MCs—but also informal, such as in personal conversations or spontaneous discussions that might arise when MCs participated in military activities, moved alongside military personnel, or shared meals with them.

Concerning *moral injury*, it was noteworthy that the vast majority were only slightly, somewhat, or vaguely familiar with the concept. Only a small number reported having a solid understanding of it. This corresponded with the observation that the majority did not employ the concept in their practice of Military Soul Care.

It should further be emphasized that ethics and morality are broad domains in a Swedish military context, extending beyond rules of engagement and conduct to encompass the full spectrum of human existence, from life to death. This includes, for example, how a military unit, when circumstances do not allow for any other solution, ensures that a fallen comrade is buried in a dignified and ritually appropriate manner, including the duties of war grave services. The Church of Sweden and its clergy, and thus also the MCs, exercise a state-authorized function in this regard, given their responsibility for burials on behalf of the Swedish state.

This brings us to the next thematic contribution, which emanates from context and culture.

### Framing cultural coherence to assist meaning-making

The third major theme in understanding the contribution of Military Soul Care, as articulated by the MCs, was the insight that they operate within a specific cultural context. In this context, they draw on forms of meaning-making that resonate with the cultural framework.

In this regard, the understanding of the Swedish cultural context becomes central, not least with reference to the role and position of the Church within Swedish society. The Church of Sweden was the state church until the year 2000 and still formally encompasses more than half of the population, while remaining relevant for an even larger part of society through its traditions, rites, ceremonies, and responsibilities for burial—elements that can be said to constitute vital parts of what is perceived as Swedish identity. The Church’s general, generically accessible, and existential approach is particularly prominent in various forms of soul care activities in Swedish society (e.g., in hospitals, prisons, schools, police, airports), and not least in Military Soul Care with its specific framework ACCES.

When MCs, through Military Soul Care, approach questions of meaning together with military personnel, this should be understood as activities embedded both in Swedish societal culture (e.g., soul care, funerals, the value foundation affirming the equal worth of all, gender equality, etc.) and in military culture (e.g., Military Soul Care conversations with absolute secrecy, the field sermon—*korum* in Swedish). Added to this are well-established church-specific practices such as baptism, wedding, communion, and theological concepts such as suffering, hope, faith, forgiveness, atonement, and growth.

Against this background, many MCs articulated how Military Soul Care contributed not only to drawing upon and sustaining a prevailing sense of coherence, but also to cultivating a deeper, even existential, understanding of the service members’ engagement in military activities.

Participant 3 (Home Guard Battalion MC) described it as:

“Contributing to the maintenance of tradition and connecting soldiers to history, the present, and the hope of the future.”

Participant 5 (Home Guard Battalion MC) emphasized that:

“It contributes to the creation of meaning and coherence.”

Participant 20 (Home Guard Battalion MC) explained:

“It is a tool for finding something to gather around beyond purely human ceremonies, where we provide a space to reflect on whether there is something greater, something higher, another world. Ceremonies show that you matter, that you are cared for in life and in death. My presence offers comfort, whether or not I say anything, and regardless of whether others believe or not. I represent some kind of steady foundation, and by this I do not mean the Church of Sweden, but something greater still.”

Participant 30 (Regiments, Air Bases, Naval Stations, and Garrisons MC) stated:

“It also provides a sense of coherence. It breaks the loneliness of individualism and places the human being within a context larger than oneself.”

Participant 32 (Regiments, Air Bases, Naval Stations, and Garrisons MC) observed:

“It contributes to an ability among those I have met to better preserve a sense of coherence despite the tasks they carry out. It contributes to an elevated capacity to defend what we are set to defend—our values, democracy, our free society.”

Participant 33 (Regiments, Air Bases, Naval Stations, and Garrisons MC) reflected:

“Through rituals and practices, and sometimes only through its presence, Military Soul Care bears witness to ‘something higher.’ It can deepen the sense of purpose, offering a language for self-sacrifice and for giving oneself for the collective and for a greater good.”

Participant 39 (Regiments, Air Bases, Naval Stations, and Garrisons MC) added:

“Military Soul Care also contributes to community, participation, meaningfulness, and the sense of belonging to something greater than ourselves, which can be especially significant in difficult and demanding situations.”

Participant 41 (Regiments, Air Bases, Naval Stations, and Garrisons MC) described it as:

“A sustaining of tradition, a framework for faith, and a support for the growth of human becoming.”

By situating meaning within a specific cultural context and frame, MCs employed a sense of coherence that proved valuable for Swedish military personnel in difficult and chaotic situations. These situations were often marked by the dissolution of coherence, tradition, and order. Yet coherence, tradition, and order themselves became a way of remembering what was being defended. They served as a means of sustaining the will to fight in order to protect and preserve the society to which soldiers belonged—a society where family, relatives, and friends had both gone before and still remained, continuing to live their lives.

The contribution and significance of Military Soul Care for the resilience, endurance, and motivation of military personnel stemmed, among other things, from the possibility of drawing on deeply rooted and timeless traditions, rites, ceremonies, and mindsets within the Swedish context. In this regard, the Church must be understood as embedded within the culture. The broad ecclesiastical toolbox, together with the MCs’ extensive experience in pastoring and providing general existential care for people in all situations of life, embodied an inherent capacity to foster meaning, cultivate purpose, and provide deeper understanding of the activities military personnel engaged in. This capacity became particularly vital in times of crisis and war, precisely because of its cultural embeddedness.

## Discussion

There are several key takeaways from this study that warrant discussion and need to be related to prior research.

### A culturally sensitive approach

MCs perform functions that mental health experts neither do nor are trained to do—namely, addressing the spiritual, religious, and existential dimensions of service members’ well-being, in accordance with the biopsychosocial-spiritual model (BPSS) (62–66). This may involve questions of faith, identity, meaning, purpose, suffering, guilt, and so on. At this general level, there is broad agreement that, within the BPSS model, MCs contribute in a broad and inclusive manner to the spiritual dimension of health in military contexts (see, for example, 3, 4, 6, 9, 11–14, 67).

However, the meaning and organization of military chaplaincy services differ significantly across countries, not least within NATO. This variation is largely due to differences in societal structures, laws, cultures, and traditions regarding religion, faith, and chaplaincy (2). For example, the Nordic countries are still to some extent shaped by former state and folk churches, which for centuries have influenced societal and cultural life and continue to do so, even though these countries are described as secular and pluralistic (68, 69). By contrast, Western European countries, where the Nordic state church model has not played the same role, display a greater degree of plurality in their chaplaincy services (e.g., representation of both major and minor religions, including secular humanists, through their own MCs).

Generally, there is no overarching standardization through checklists, diagnostic criteria, or evidence-based treatment methods across chaplaincy services. This is primarily because chaplains operate within a different ontology (e.g., transcendence, sacraments) and epistemology (e.g., Bible, scriptures, hermeneutics), rendering such medical approaches inadequate and incompatible (4, 11).

Altogether, these are among the reasons why NATO member states bear primary responsibility for their own chaplaincy services. A deep understanding of a country’s society, culture, and traditions is essential for understanding its chaplaincy service. This implies that it is of paramount importance to develop a culturally sensitive approach upon entering the field of military chaplaincy.

### Advancing the dialogue between theory and empirical research

The Swedish model rests on an almost 500-year uninterrupted tradition of priests from the Church of Sweden practicing Military Soul Care, now formalized within the ACCES framework (11). The Swedish Armed Forces and the Church of Sweden, as two longstanding societal institutions, have coexisted for centuries, cultivating a partnership that effectively supports the enhancement of military capability (44). This is achieved by addressing the existential dimension in ways that contribute to a sense of coherence, spiritual balance, existential health, mental resilience, and moral fortitude (53). This constitutes the descriptive theory underpinning the understanding of the contribution of Military Soul Care.

However, when MCs themselves are asked to articulate their contributions, they emphasize themes such as 1) preserving the human character in inhuman situations, 2) sustaining ethical and moral dimensions and reflections in a military context, and 3) framing cultural coherence to assist meaning-making in a military context. The final theme, in particular, shows a strong and direct affinity with Antonovsky’s (70) original approach to sense of coherence (SOC) and meaning-making. Antonovsky (70) divided the meaning-making dimension of SOC into three subcomponents: comprehensibility (i.e., the ability to understand events in a rational way), manageability (i.e., the availability of resources to handle a situation), and meaningfulness (i.e., the capacity to create emotional meaning and existential motivation in a situation). The analysis in this study resonates primarily with the dimension of meaningfulness and the existential aspects of SOC.

While the findings of the themes do not contradict the descriptive theory of Military Soul Care, which is grounded in practical experience, they suggest that these three thematic emphases deserve particular attention when articulating the framework’s contribution.

### War and the human character

As noted earlier, recent research from the ongoing war in Ukraine has shown that a significant component of wartime military chaplaincy consists in safeguarding the moral character and compass of military personnel (26, 27). This is because the relentless brutality of war continuously erodes the moral frameworks that previously structured civilian identity and everyday life, frameworks that collapse in the wartime context. Ukrainian MCs have emphasized the importance of tirelessly working with morality and ethics to counteract this erosion of soldiers’ character, which otherwise risks impairing their operational capacity and leading to poor judgment, recklessness, and wartime transgressions that may prove unbearable in the long term (26, 27).

In light of the present study’s chorus of voices emphasizing the preservation of human character, humanity, and human dignity, together with the sustaining of ethical and moral frameworks as core contributions and deeper meanings of Military Soul Care, Swedish MCs resonate strongly with the hard-won experiences of their Ukrainian colleagues. This orientation reflects a more broadly human or existential understanding rather than an explicitly religious or spiritual one—leading to the next point of discussion.

### An all-encompassing existential rather than spiritual/religious approach

In a Swedish context, the concepts of *spiritual* and *religious* are partly problematic, as they do not resonate well with culture or everyday language (11). *Spiritual* is primarily a church-based term without a general cultural grounding. *Religious* tends to be a categorical label used by scholars, but it is not commonly employed as a self-description in everyday life. By contrast, the term *existential*— referring to life and existential questions concerning meaning and purpose—resonates far more strongly with how Swedish MCs approach service members in an all-encompassing manner within Military Soul Care (11).

Closely aligned with the existential approach endorsed by MCs, recent work on existential health by the Swedish Public Health Agency (71) points to a broadly shared cultural understanding of this perspective, despite the absence of a single, definitive definition. Key existential dimensions of health in contemporary Sweden include experiencing meaning in life; relating to oneself, others, and nature; maintaining a sense of coherence and safety; and perceiving oneself as part of something greater, with the Church likewise identified as a significant actor in addressing the existential dimension of human life (71).

Of course, rituals and traditions deeply embedded in Swedish and military culture—such as the field sermon—are also used to situate people within a sense of coherence. In the sociology of religion, the concept of *cultural Christians* is often used to describe Swedes’ relationship to the state church they grew up with, where they were socialized into traditions and rituals embedded in Swedish society over centuries (41). The term underscores knowledge of ritual and tradition primarily from an existential rather than a strictly theological standpoint (11). In everyday life, people may not rely heavily on the church, but in life’s decisive moments—death, grief, crises, and catastrophes—many turn to the church to meet their existential needs (43, 72, 73).

### The potential for advancing the understanding of moral injury

Recent research has increasingly emphasized that chaplains possess expertise and unique competencies for addressing *moral injury*. A growing body of literature has highlighted the value of pastoral experience and theological or ritual approaches for dealing with moral injury related to betrayal, transgression, guilt, and shame—concepts often associated with acceptance, forgiveness, reconciliation, and restoration (3, 7, 12, 13, 15, 16, 67, 74).

Among the 50 MCs in this study, however, knowledge of *moral injury* was limited, and the concept was not directly employed within the framework of Military Soul Care. While the concept of moral injury is certainly appealing from existential, theological, and chaplaincy perspectives, it is perhaps unsurprising that it has not yet gained deeper theoretical or practical traction in the Swedish context. In the empirical material, participants reported various ethical and moral challenges embedded in the complex situations they encountered. However, even if a different research design—such as interviews—had been employed, it would still be difficult to extract information about the individual-specific nature of moral trauma or injury, as the absolute confidentiality maintained by MCs prevents them from disclosing such matters at the individual level.

It is worth recalling that the term *moral injury* was originally coined in a psychiatric context during the 1990s (60, 61), and for the first fifteen years it remained largely confined to psychological treatments within veteran care (75). Theology and soul care, together with clerical education in Sweden, have resisted the psychologization that characterized much of late 20th-century practice (50, 51), and continue to do so both in priestly training and in broader approaches to soul care. This does not, however, preclude the possibility that *moral injury*, which is now widely discussed among MCs worldwide, could be compatible with Military Soul Care and deepen understanding of what occurs when humanity is lost or when moral and ethical boundaries are transgressed.

Combined with the absolute secrecy guaranteed in Swedish chaplaincy—contrasting with mandatory reporting requirements in military psychiatry and psychology—this creates highly favorable conditions for integrating *moral injury* into a Swedish context. Thus, competence-building around the concept and its practical use would likely be beneficial for Swedish MCs in their practice of Military Soul Care.

Moreover, this explicitly connects with a recent development in the field of moral injury, which highlights the character domain—specifically character failure—as a potential source of moral injury (17). This is particularly relevant to the theme of war and the human character, as discussed above in relation to the well-articulated theme of preserving the human character in inhuman situations.

### Conclusion and recommendations

This study demonstrates that MCs make essential contributions within the biopsychosocial-spiritual (BPSS) model by addressing existential and moral dimensions of service members’ well-being—areas that lie beyond the conventional parameters of psychiatric intervention. The findings align with prior research (62, 64, 66), which emphasizes that existential meaning, moral coherence, and spiritual orientation are central to resilience, psychological stability, and recovery.

The Swedish model illustrates how chaplaincy is culturally and institutionally embedded, indicating that chaplaincy services must be tailored to national or culturally similar contexts rather than standardized across systems. Swedish MCs frequently conceptualize distress in existential rather than religious or medical terms, reflecting how many service members understand suffering, identity disruption, and moral tension.

Recent developments in psychiatric literature on moral injury provide an important framework for interdisciplinary collaboration. However, the increasing dominance of this concept risks narrowing the understanding of existential distress. Alternative constructs, such as spiritual injury, spiritual struggle, or existential struggle, may more accurately reflect the diversity of experiences both within and across military cultures and subcultures. Maintaining conceptual plurality is therefore essential to fully understand the complexity of service members’ well-being.

The recommendations are as follows:

- Support the development of culturally informed chaplaincy models that preserve the integrity of national traditions and avoid externally imposed standards.
- Enhance competence-building around moral injury while considering additional constructs, such as spiritual injury or struggle, to ensure a broader understanding of existential distress relevant to diverse populations.
- Strengthen dialogue between empirical psychiatric research and chaplaincy-based descriptive theory to refine and advance the BPSS model in ways that reflect both practice and evidence.
- Promote the continued development of existential frameworks as distinct from current medical, spiritual, or religious models.
- It is important to recognize the asymmetry in institutional authority between medicine and chaplaincy; while psychiatry holds clinical power, chaplaincy provides essential existential resources that contribute meaningfully to holistic care.

In conclusion, the BPSS paradigm supports interdisciplinary collaboration that preserves disciplinary integrity. A pluralistic and culturally grounded approach offers a more comprehensive path to improving mental health outcomes in military populations.

## Limitations and future research

A study of this nature inevitably carries methodological limitations. It can never be as in-depth and exploratory as an interview study, and because the questions were open-ended, the data does not lend itself readily to statistical analysis. Nevertheless, it provides valuable insights into participants’ thoughts and perspectives, collected anonymously, and in this case representing nearly 50% of the existing population of MCs in the Swedish Armed Forces. This suggests that a substantial proportion of the population is likely to recognize themselves in the responses, even though the analytical thematic process in some instances extended beyond what individual respondents may have intended regarding the contribution and meaning of Military Soul Care.

At the same time, it would in certain cases have been desirable to explore responses in greater depth, something that was unfortunately not possible within the present research design. For this reason, a key theme for future research is the use of qualitative interviews to gain a deeper understanding of the work of preserving the human character, and of what constitutes and characterizes this work beyond its engagement with moral and ethical concerns situated within a particular cultural context.

Another limitation is that the study is confined to the profession’s own perspective—namely, the MCs’ self-understanding of the contribution of their work—which was the study’s intended focus. This perspective does not necessarily reflect the Armed Forces’ institutional understanding of the practice, even if the findings clearly resonate with the Armed Forces’ broader understanding and purpose of Military Soul Care (53).

From an international perspective, further research is strongly encouraged that adopts a culturally sensitive approach to military chaplaincy. Such research would provide a deeper illustration and unpacking of how society, culture, tradition, and legal frameworks interact with and shape the services provided by MCs.

## Data Availability

The original contributions presented in the study are included in the article/supplementary material. Further inquiries can be directed to the corresponding author.
